# The Structural and Mechanical Basis for Passive‐Hydraulic Pine Cone Actuation

**DOI:** 10.1002/advs.202200458

**Published:** 2022-05-14

**Authors:** Carmen J. Eger, Martin Horstmann, Simon Poppinga, Renate Sachse, Rebecca Thierer, Nikolaus Nestle, Bernd Bruchmann, Thomas Speck, Manfred Bischoff, Jürgen Rühe

**Affiliations:** ^1^ Department for Microsystems Engineering Chemistry and Physics of Interfaces University of Freiburg Freiburg 79110 Germany; ^2^ Cluster of Excellence livMatS @ FIT – Freiburg Center for Interactive Materials and Bioinspired Technologies University of Freiburg Georges‐Köhler‐Allee 105 Freiburg im Breisgau 79110 Germany; ^3^ Plant Biomechanics Group Botanic Garden University of Freiburg Schänzlestraße 1 Freiburg im Breisgau 79104 Germany; ^4^ Department of Biology Technical University of Darmstadt Botanical Garden Schnittspahnstraße 2 Darmstadt 64287 Germany; ^5^ TUM School of Engineering and Design Department of Engineering Physics and Computation Technical University of Munich Boltzmannstraße 15 Garching b. München 85748 Germany; ^6^ Institute for Structural Mechanics University of Stuttgart Pfaffenwaldring 7 Stuttgart 70550 Germany; ^7^ BASF SE Carl‐Bosch‐Strasse 38 Ludwigshafen am Rhein 67056 Germany; ^8^ Present address: Department of Animal Ecology Evolution and Biodiversity Ruhr University Bochum Universitätsstr. 150 44801 Bochum Germany

**Keywords:** µ‐CT scans, finite element simulation, hydration measurement, kinematical and structural analysis, model for water absorption, pine cone movement, tissue mechanics

## Abstract

The opening and closing of pine cones is based on the hygroscopic behavior of the individual seed scales around the cone axis, which bend passively in response to changes in environmental humidity. Although prior studies suggest a bilayer architecture consisting of lower actuating (swellable) sclereid and upper restrictive (non‐ or lesser swellable) sclerenchymatous fiber tissue layers to be the structural basis of this behavior, the exact mechanism of how humidity changes are translated into global movement are still unclear. Here, the mechanical and hydraulic properties of each structural component of the scale are investigated to get a holistic picture of their functional interplay. Measurements of the wetting behavior, water uptake, and mechanical measurements are used to analyze the influence of hydration on the different tissues of the cone scales. Furthermore, their dimensional changes during actuation are measured by comparative micro‐computed tomography (µ‐CT) investigations of dry and wet scales, which are corroborated and extended by 3D‐digital image correlation‐based displacement and strain analyses, biomechanical testing of actuation force, and finite element simulations. Altogether, a model allowing a detailed mechanistic understanding of pine cone actuation is developed, which is a prime concept generator for the development of biomimetic hygromorphic systems.

## Graphical abstract

This contribution describes the hygroscopic opening and closing mechanism of pine cones in terms of mechanical and hydraulic properties of the individual seed scales. Through a combination of mechanical, structural, and chemical analyses, a mechanistic model of pine cone actuation and hydraulics is proposed.

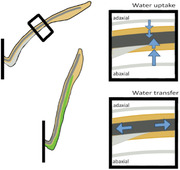

## 1 Introduction

**Figure 1:**
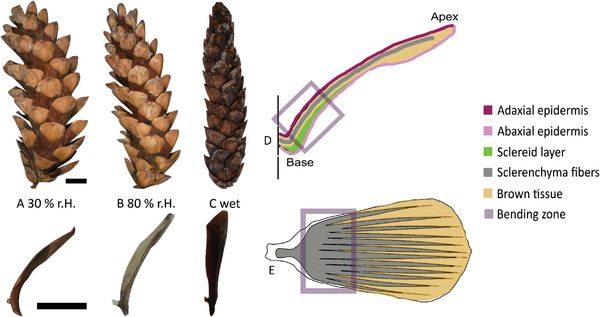
The *P* *inus* *wallichiana* cone at different levels of humidity. Open cone at 30% relative humidity (rh, at 23 °C) and the corresponding bent shape of the scale in lateral view A). After 7 h at 80% rh (at 23 °C), the cone is slightly closed. The single scale shows a slight bending toward the cone axis B). A cone soaked in water will close completely. The individual scale is completely straight upright C). Schematic representation of a scale and its underlying architecture D,E). Longitudinal section. The adaxial epidermis (dark purple) and the abaxial epidermis (light pink) envelop the sclereid cells (green) and the sclerenchyma fiber strands (gray), which is embedded in a matrix of brown tissue (light brown) D). Top view on a scale. The sclerenchyma fibers extend as strands (gray) into scale periphery and are embedded by brown tissue (light brown). In the bending zone, almost no brown tissue can be found. Here, the structure can be considered as a functional bilayer E). Scale bars: 2 cm.

Female cones of the genus *Pinus* (Pinacae) with their spirally arranged seed scales are prime examples for structures executing passive nastic plant motion.^[^ ^1^, ^2^ ^]^ Under wet environmental conditions, when seed release for wind dispersal is unfavorable, each scale on the cone is bent toward the cone axis and protects the seed. Under dry environmental conditions, when seed dispersal by wind is favored, the scales bend away from the cone axis and the seeds are released (**Figure** **1**A–C). The transition from the “wet state” to the “dry state” causes the passive, water‐driven bending motion of the individual scale, which is dictated by the different mechanics and swelling/shrinking properties of the tissues.^[^ ^2^, ^3^, ^4^, ^5^ ^]^ An abaxial sclereid layer swells and shrinks in longitudinal direction, as dictated by the cellulose microfibrils embedded in the cell walls.^[^ ^3^ ^]^ A (more adaxially positioned) sclerenchymatous fiber layer is assumed to be the passive, resistance layer, hereby determining the bending deformation.

According to the literature, the seed scale functions similar to a bimetallic strip^[^ ^6^ ^]^ but responds to humidity instead of heat due to its functional bilayer architecture. However, this bilayer setup is only present in the very basal part of the scale, where the actuating layer (sclereids) and resistance layer (sclerenchymatous fibers) are densely packed and form more or less continuous layers. As previous studies showed,^[^ ^1^, ^7^ ^]^ this widely accepted construction plan is rather simplistic regarding the whole scale, as it does not incorporate several structural features additionally present: 1) the sclerenchymatous fibers do not form a continuous tissue layer, but extend as strands into the scale periphery; 2) the sclerenchyma fiber strands are embedded in a matrix of the so‐called brown tissue; and 3) the scale is enveloped by the epidermis, which, as the outermost scale tissue, is in direct contact with the environment and governs the uptake and release of humidity (Figure 1D,E). The mechanical contribution of these tissues to the motion, as well as their behavior during water absorption and desorption is not known so far.

For the scale motion to take place, water molecules must diffuse through the epidermis. They deposit onto surfaces either as fog or dew.^[^ ^8^ ^]^ While fog consist of small water droplets (with diameters of around 10 µm) that are already in the air and can become deposited on various surfaces, dew only develops when the saturation point is reached and humidity condenses into water droplets. However, besides super saturation of the air or quick temperature changes other factors for dew formation on surfaces have to be taken into account as well. This can include chemical properties, like hydrophilicity, and/or the roughness of the surface, as known from the elytra of the desert beetle *Physasterna cribripes*, which cause dew accumulation and formation of a water drop in the moist air in the desert at night.^[^ ^8^ ^]^ However, how in detail the pine cone achieves water uptake from the environment and, consequently, distribution within the scales is completely unknown so far.

The pine cone bending is driven by humidity changes and does not mandatorily require direct contact with water. The diffusion of water molecules generally depends on temperature, time, and material properties, like hydrophilicity or porosity.^[^ ^7^ ^]^

A recently published paper by Quan et al. investigated the tissues and the bending motion of *Pinus torreyana* cone scales by scanning electron microscopy (SEM) and µ‐CT.^[^ ^9^ ^]^ They found that the sclereid layer is characterized by a porosity gradient, where the highly porous part of the sclereid layer acts as a cushion between the sclerenchyma fiber strands and the lesser porous sclereid layer.^[^ ^9^ ^]^ Thus, they developed a tri‐layer setting for mechanical description of the bending motion, which is known also for tri‐metal strips.^[^ ^10^ ^]^ Furthermore, the motions of the individual tissues were investigated. In this paper, we present our complementary results to this with more details on the physic‐chemistry of water uptake and its influence on scale motion and tissue (de)swelling. The water uptake and distribution throughout the scales of *P. wallichiana* is analyzed and linked to the changes of mechanical and dimensional changes of its tissues and finally to the global movement response.

## 2 Experimental Section

### 2.1 Kinematic and Structural Analyses

#### 2.1.1 Sample Source and Preparation

One cone of *Pinus sylvestris* (for µ‐CT analyses) and seven cones of *Pinus wallichiana* (for all other experiments) were obtained from the collection of the Botanic Garden Freiburg. Unless otherwise specified, scales were detached from the cone and used for the respective experiments. Several experiments (structural analysis via SEM, kinetic and gravimetric water uptake, mechanical measurements, contact angle measurements, force measurements) required separation of the individual scale tissues. Accordingly, scales from the middle parts of the cones were placed in distilled water at room temperature overnight. In the wet state, samples of the adaxial and abaxial epidermises, sclereids, sclerenchyma, and brown tissue could be separated by a scalpel for further testing.

#### 2.1.2 Motion of a Whole Scale

Bending responses of 12 scales from four pine cones of *Pinus wallichiana* (P. wallichiana) were recorded with the time lapse mode (1 picture/2 s) of a GoPro Hero 7 black camera (GoPro Inc., San Mateo, CA) inside a climate chamber (Binder, KBF 115). The relative change of the angle of the tip of the scale was measured as function of relative humidity (rh), which was increased from 30% to 80% rh (and in reverse afterward) at 23 °C in 10% intervals. The scales were allowed to swell to equilibrium for over 7 h. This equilibrium time was determined beforehand by measuring the time until the scale did not bend any further. Afterward, gravimetric measurements of the water uptake within 7 h intervals and 10 h intervals were performed. Within 7 h, however, the scale already reached the equilibrium, since its weight did not increase any further. The bending response was quantified with the software ImageJ. A graph paper was used as scale.

For analyzing water uptake, scale bending responses with adaxial, abaxial or both sides coated was measured as described above. The before used 12 scales were divided into two groups of 6 each, which were first coated on the adaxial or abaxial side, respectively, before all scales were coated on the remaining uncoated side. The coating was conducted with poly(methyl methacrylate) (PMMA) dissolved in acetone (*c* = 100 mg mL^−1^). The polymer solution was applied to the appropriate side(s) with a small sponge and allowed to dry completely before the measurement so that a few µm thickness was obtained. The thin, hydrophobic and (due to the low thickness) flexible PMMA coating prevents water penetration without mechanically hindering the scale motion.

#### 2.1.3 Structural Analysis via SEM

Dry tissue samples, prepared as stated above from one scale of one pine cone, were sputter‐coated with a few nanometer thick gold‐layer (Sputter Coater 108 auto, Cressington Scientific Instruments Ltd., Watford, England) and observed with a scanning electron microscope (SEM) LEO 435 VP (Leica, Wiesbaden, Germany). The acceleration voltage was 15 kV, the chamber pressure was in the range of 10^−5^ bar.

#### 2.1.4 Structural Analysis via Optical Microscope

Four scales from the middle part of one cone of *P. wallichiana* were removed and placed in distilled water at room temperature overnight. For gaining tissue thickness profiles, two of the wet scales were cut longitudinally and two were cut laterally in 0.5 cm wide stripes with a scalpel. The thicknesses of the sclereid and sclerenchyma layers were measured with a microscope (BX51, Olympus, Tokyo, Japan) with 20x magnification.

### 2.2 Hydration Experiments

#### 2.2.1 Kinetic of Contact Angle Measurements

Contact angle measurements (OCA20 setup from Dataphysics GmbH) were performed for quantifying the wetting behavior of 5 µL droplets of deionized water on the abaxial and adaxial epidermises, sclereids, sclerenchyma, and brown tissue over time. Four scales from two different cones were used to prepare the samples. To obtain flat samples, the surface of the sclereids, sclerenchyma, and brown tissue was smoothed with a scalpel and dried under a metal weight (250.2 g), while the epidermis was just dried under a metal weight. This method is expected to not influence the surface roughness of the epidermis. The tissue with the sclerencyma fibers was placed such that it was oriented perpendicular to the optical axis of the instrument and fixed with double‐sided tape onto the glass slide to ensure that it does not move. The static contact angles were measured in small intervals as long as the contact angle decreased rapidly visually. Intervals were increased to around 50–60 s when the contact angles only decreased slowly. The measurements were performed until the respective droplet had either vanished (due to evaporation and/or absorption) or until the contact angles remained constant. A droplet on a standard microscopy glass slide was measured as a reference to distinguish evaporation from absorption.

#### 2.2.2 Gravimetric Water Uptake

Each tissue type was placed in a climate chamber (Binder, KBF 115) and weighted with a balance (Sartorious QUintix224‐1S, 0.1 mg accuracy) across 30–80% rh (at 23 °C) in 10% intervals with 1 h waiting time per interval. The balance logged the weight every minute. The sample of the sclereid cells consisted of two rectangular cuts from the base (where the sclereid layer is the thickest) of two different scales with a combined weight of 92.7 mg. The biggest pieces of the brown tissue of up to three scales were used for this measurement, since it was not possible to prepare one single big piece of brown tissue. One sclerenchyma fiber bundle from one scale was measured (122.0 mg) and again with another approx. half bundle (155.6 mg) from another scale.

In addition, whole scales and dissected tissues adaxial of the sclerenchyma (adaxial epidermis + brown tissue) and abaxial of the sclerenchyma (abaxial epidermis + sclereid), as well as the sclerenchyma itself were submersed in water. The weights of the tissues before and after hydration were measured to obtain the water uptake. The weights after hydration were determined after carefully removing excess water from the respective surfaces using paper towels and waiting an extra minute to let remaining water on the surface evaporate.

### 2.3 Tissue Mechanics and Dimensional Changes During Actuation

#### 2.3.1 Mechanical Properties of Tissues

Young's moduli of the individual scale tissues were determined with an atomic force microscope (AFM) (Nanowizard4 from Bruker Nano GmbH, Berlin, Germany). The scale tissues were separated as described in chapter “sample source and preparation” above. The samples were smoothed with a scalpel and swollen to equilibrium in 80% rh (at 23 °C) overnight. For each measurement one sample from one cone scale was used and measured on an area of 625 µm^2^ with 64 points twice. This measurement was repeated at each humidity level. A wet microfiber cloth was hanged into the chamber of the AFM and a small ventilator was placed in front of the cloth. The water in the cloth evaporated overnight and increased the relative humidity to 80%. Microindentation was conducted with a sphere AFM tip (CP‐NCH‐SiO‐C, Ø 6.62 µm SiO particle, force constant 10–130 N m^−1^, resonance frequency 204–497 kHz, from NanoAndMore GmbH, Wetzlar, Germany), while the rh was decreased from 80% to 30% by slowly blowing nitrogen into the chamber (at 23 °C) over 9 h. All tissues were indented perpendicular to the longitudinal axis of the scale. The indentation force was set to 1200 nN for the sclerenchyma fibers strands, 1000 nN for the sclereid layer and 800 nN for the brown tissue. The indentation depth was 130–150 nm for the brown tissue, 200–270 nm for the sclereid tissue and 30–65 nm for the sclerenchyma fiber strands. For the brown tissue and sclereid tissue, the Young's moduli were measured each percent rh, since they reached swelling equilibrium fast. The Young's modulus of the sclerenchyma fiber strand was measured in 5% rh intervals. The Young's moduli of the adaxial and abaxial epidermis could not be measured because the rough surface interfered with the measurement.

#### 2.3.2 Simulation

Simulations of the bending deformation were performed using ANSYS (Release 18.0, ANSYS Inc., Canonsburg, USA). They are based on finite element models that are analyzed assuming geometrically nonlinear quasi‐static behavior, as no inertia effects, such as vibrations, were expected. The geometry was idealized from an average pine cone scale as illustrated in Figure 1 and was discretized by eight‐node hexahedral finite elements (SOLID185). The structure is fixed at the very proximal end of the scale. In the bending zone, a fine mesh is used in order to accurately resolve the deformation. The rest of the scale is discretized with a much coarser mesh as this part does not contribute to the mechanical response. It is rather introduced for postprocessing reasons, i.e., to measure the resulting angle due to bending and to compare the simulation results to experimental measurements. Furthermore, linear elastic material models with varying Young's moduli for the respective layers and a Poisson's ratio of *ν* = 0.3 was applied. As only strain in longitudinal direction was expected, no anisotropic material behavior was taken into account. The Young's modulus of the sclereid layer varies according to the experimental values (Figure 8), which were approximated by a linear regression between 21 and 37 MPa. On the other hand, Young's modulus of the sclerenchyma fiber strands was characterized by large standard deviations and, therefore, used as order of magnitude but not set. In this way, the measurement accuracy could be supported by simulation. For the actuation, a temperature load case was applied. This load case simulated the desiccation of the active layer with a volumetric shrinking due to a temperature decrease. This shrinking occurred in the longitudinal direction of the scale.

#### 2.3.3 Force Measurements

For measuring the forces generated by whole scales as well as dissected tissues and combinations thereof, samples were tightly clamped at their base and placed beneath a stationary force sensor (static load cell, +/− 100 N, Instron, Darmstadt, Germany). The scales’ tips/apical tissue ends touched the force sensor from the beginning of the experiment onward. Two cones were used to prepare the samples. For the analysis, five undissected scales were used, four scales for the abaxial and adaxial tissue, five scales for the abaxial and sclerenchyma tissue and six scales for the adaxial and sclerenchyma tissue. For wetting‐induced actuation, the scales as well as a plate attached to the sensor were submersed in water using a small water tank. Samples touched the measuring plate centrally and at an angle of about 90°. The length of the scales was ≈4.5 cm. During drying‐induced actuation, scales were clamped the other way around to measure the forces exerted in the opposite direction. The contributions of the single tissues to the global scale movement and force generation could be evaluated by measuring 1) whole scales, 2) only adaxial epidermis with brown tissue, 3) only the abaxial epidermis with sclereid layer, 4) only sclerenchyma fiber strands, and 5) combinations of sclerenchyma fiber strands with adaxial epidermis and brown tissue and 6) sclerenchyma fiber strands with abaxial epidermis with sclereids. Apart from using the tissues in the quantity of actual scales, force generation was also determined per gram dry weight of the samples investigated.

#### 2.3.4 Micro‐CT Scans

For analyzing tissue dimensional changes, µ‐CT‐scans of a few millimeter long seed scale of *P. sylvestris* in the wet and dry state were conducted. These scans were taken with a Bruker Skyscan 1172 (Bruker Micro‐CT S.A. Kontich, Belgium) at 40 kV and a current of 250 µA Images were acquired with a pixel resolution of 6 µm (wet), respectively, 9 µm (dry). The acquired stacks were resized to the size containing information on the scale using Fiji.^[^ ^11^ ^]^ These cut stacks were imported into 3D Slicer^[^ ^12^ ^]^ (version 4.11,) and every 20th to 30th slice was manually labeled for the occurrence of sclerenchyma 1), sclereids 2), brown tissue 3), and other 4). Differentiation of the epidermises was not possible. Consequently, the stack with the labeled slices was exported and the image, as well as the label stack, was also uploaded to biomedisa.de.^[^ ^13^ ^]^ This open‐source online platform for semiautomatic segmentation uses weighted random walks for smart interpolation, which is a drastic improvement in accuracy compared to purely interpolation‐based semiautomatic segmentation. The completely segmented stacks were then downloaded and again imported into 3D Slicer. All tissues were separately converted to stl‐models and saved. These models were then exported and colorized using Meshlab.^[^ ^14^ ^]^ Measurements of thickness and length were conducted using Meshlab and Blender (version 2.83, Blender Foundation, Blender Institute Amsterdam, https://www.blender.org/, 2020).

#### 2.3.5 3D Digital Image Correlation (DIC)

Displacement and strain of scale surfaces of *P. wallichiana* were analyzed with the 3D digital image correlation (3D‐DIC) method.^[^ ^15^, ^16^ ^]^ Nine scales from two different pine cones were used for this measurement. Accordingly, the scales were submersed in water overnight, the excess water was removed superficially using paper towels, and the surface was covered with chalk spray (3D Laser Scanning Spray, Helling GmbH, Heidgraben Germany). On this whitened surface, a stochastic speckle pattern was carefully applied using black spray paint (Liquitex Professional, carbon black, ColArt, Le Mans, France). Using a stereo camera setup including two USB‐3.0 PixeLink cameras (PL D685CU, pixel size 4.8 µm), equipped with 100 mm lenses (Makro‐Planar 2/100 ZF, Carl Zeiss AG, Oberkochen, Germany), time lapses were recorded with one image every 30 s for 7 h, covering the whole drying‐induced movement processes. The deformation and strains on the abaxial and adaxial surfaces of 9 scales in total were analyzed with the software Aramis 2016 (GOM GmbH, Braunschweig, Germany), which was also used to calibrate the system.

#### 2.3.6 Statistical Analysis

Statistics for the force and total water uptake measurements were carried out using the software R.^[^ ^17^ ^]^ As data were non‐normally distributed, a Kruskal–Wallis‐Test was used to test for significant differences. Differences between tissues were examined using the Dunn's‐Test from the R package PMCMR,^[^ ^18^ ^]^ as posthoc‐analysis. “Tukey” as method and “fdr” for the p‐adjustment of this test was used. All tests were conducted to an alpha‐value of 0.05 (*). The sample sizes of all experiments are given in Tables S0–S1 (Supporting Information) and in the Experimental Section of each experiment. Data were not preprocessed in any kind. Data representation shows boxplots with median, 2nd and 4th quartiles as well as whiskers representing data points within the 1.5 x interquartile range, if not indicated otherwise.

## 3 Results

### 3.1 3D Scale Architecture and Tissue Dimensional Changes During Actuation

**Figure 2:**
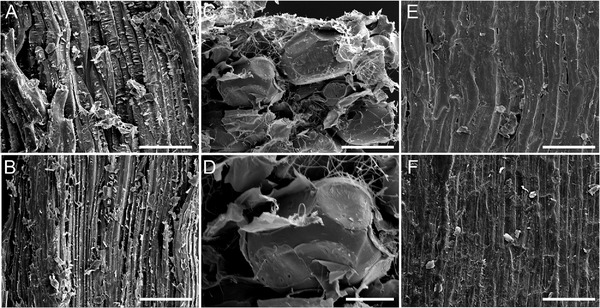
SEM figures of the sclereid, the sclerechyma fiber, brown tissue and a zoom in on one cell of the brown tissue, the adaxial and abaxial epidermis from one scale of *P. wallichiana*. The active layer consists of sclereid cells A). The sclerenchyma strand is a bundle of small fibers packed closely and oriented along the strand B). The brown tissue shows a friable structure of isodiametric cells C,D). The adaxial epidermis E) appears to be smoother than the abaxial epidermis F). The scale bars in (A,B,C,E,F) are 200 µm. The scale bar in (D) is 80 µm. The samples in (A,B,E,F) were positioned in a way that the top and bottom are the tip and base of the scale, respectively. The sclerenchyma fibers, respectively, fiber bundles and sclereid cells are thus aligned from tip to base.

The *P*. *wallichiana* seed scale consists of sclerenchyma fiber strands, sclereid cells, and brown tissue, which are encapsulated by the adaxial and abaxial epidermises. The sclereid cells consist of elongated cells (**Figure** **2**A), while the individual sclerenchyma strand is a tightly packed bundle of many small fibers (Figure 2B). The brown tissue consists of isodiametric cells (Figure 1C,D). The adaxial epidermis appears rather smooth (Figure 2E), while the abaxial epidermis shows a more structured surface in the scanning electron microscope (SEM) investigation (Figure 2F).

**Figure 3:**
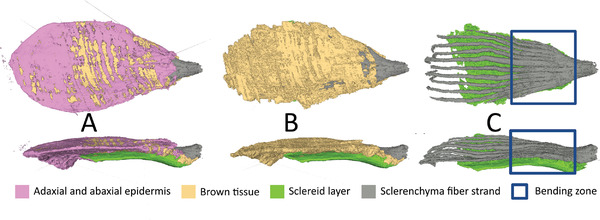
µ‐CT pictures from the top and the side of the scale of *P. sylvestris*. Entire scale with epidermis A). The sclereid layer (green), the sclerenchyma fiber strands (gray), and the brown tissue (light brown) are shown B). Only the sclereid layer (green) and the sclerenchyma fiber strands (gray) on top are shown C). The blue rectangle shows the bending zone.

We reconstructed the 3D architecture of the entire *P. sylvestris* scale and found that the scale is enveloped by the abaxial and adaxial epidermises (**Figure** **3**A). The abaxial epidermis could not be distinguished from the sclereid layer. The brown tissue is located directly under the adaxial epidermis and constitutes the largest tissue part. The apophysis is located at the scale apex (Figure 3B). The sclerenchyma fiber strands are embedded in the brown tissue; therefore, the sclerenchyma fiber strands are only visible if the brown tissue is removed. The sclerenchyma fiber strands start as one big strand at the base of the scale and then divide into many strands toward the apex (Figure 3C).

**Figure 4:**
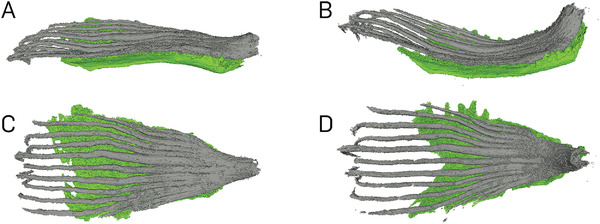
Tissues of a scale of *P. sylvestris* reconstructed based on µ‐CT‐scanning. In the dry state, the lateral view shows a straight scale, where the sclerenchyma fiber strands (gray) lies on the sclereid layer (green) A). The lateral view of the scale in the wet state shows a bent scale in upwards direction B). The top view of the dry state shows the sclerenchyma fiber strands spreading in the scale C). In the dry state, the differentiation between sclereid layer and brown tissue was not always possible, which is why the sclereid layer looks so different from C) to D). The top view of the wet state D).

The µ‐CT analyses allowed a comparison of the 3D tissue dimensions between the dry and wet states of the scale. The angular difference of the *P. sylvestris* scale in the dry and in the wet state is *γ*_water_ = 24° (**Figure** **4**A,B), while the angular difference of the *P. wallichiana* scale from 80% to 30% rh at 23 °C is *γ*_80%rh_ = 12°. The top view (Figure 4C,D) shows that the sclerenchyma fiber strands extends as strands into the scale periphery. In the wet state, the sclereid layer was 27% thicker and 22% longer in the bending zone as compared to the dry state. The sclerenchyma fiber strands were 3% thinner and on their adaxial side 1% longer on average. This is in accordance with the shrinking of the adaxial scale surface during wetting (−1% across the whole length and even −12% in the basal region). In contrast, the abaxial surface elongates during wetting (+14% across the whole length and +18% in the basal region). In terms of volume, the sclerenchyma fiber strands increased its volume by 11% during wetting, also the sclereid showed a 16% increase. The volume of brown tissue even increased by 33% upon swelling.

**Figure 5:**
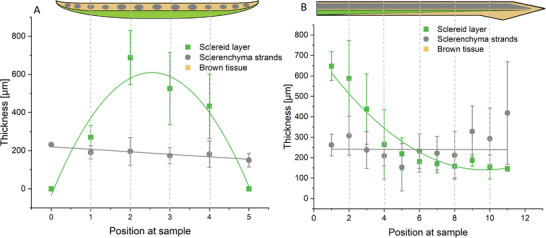
Schematic thickness profile of the sclereid layer and the sclerenchyma fiber strands from *P. wallichiana* scales. The sclereid layer (green) is the thickest in the middle of the scale and decreases toward the periphery of the scale. The sclereid layer is about 1000 µm thick at the thickest part. Across the cross‐section of the base, the thickness of the sclerenchyma fiber strands (gray) is constant and around 500 µm A). The sclereid layer (green) decreases nonlinearly toward the scale apex and the thickness of the sclerenchyma fiber strands is constant through the scale (gray) as can be seen in longitudinal section B). The different lengths of the strands over the width of the scale lead to a rather inaccurate measurement at the scale apex. The indicated thickness values are taken from ref. [15]

**Figure** **5**A,B shows the cut surface of the cross‐section and the longitudinal section of a scale. The course of the layer thicknesses is indicated in the scheme below. The sclereid layer is the thickest in the middle and the base of the scale and decreases toward the periphery and the apex. In both directions, the thickness of the sclerenchyma fiber strands stays more or less constant as mainly the thicker central sclerenchyma fiber strands remain distally.

### 3.2 Hydration Measurements

**Figure 6:**
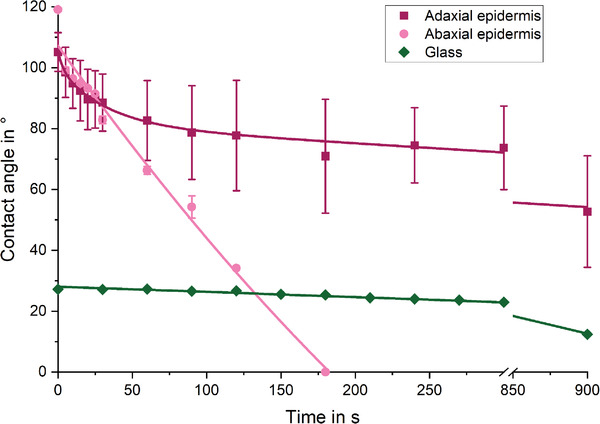
Contact angles over time on adaxial and abaxial epidermises from *P. wallichiana* scales. The contact angles of 5 µL water drop on both epidermises started around 105° to 120° and decreased in a comparable manner within the first 30 s to 90°. After 30 s, the abaxial epidermis (pink) showed a faster water uptake and absorbed the drop completely within 160 s. The adaxial epidermis (purple) showed after 60 s a similar contact angle decrease as the reference measurement on glass, which represents only evaporation and no absorption in this environment (dark green) *n* = 3. Given are mean values with standard deviation (whiskers).

The contact angles of sessile water drops were measured as a function of contact time both on the adaxial and abaxial epidermises. The measurements revealed that both surfaces are initially quite strongly hydrophobic (Θ_AD,0s_ = 105 ± 6°; Θ_AB,0s_ = 119 ± 1°) (Figure 5, pink and purple curve). After around 30 s, the contact angle decreased below 90° for both samples. On the abaxial epidermis the 5 µL water drop spreads completely within 180 s in an almost linear manner and becomes completely absorbed. The course of the decrease of the contact angles of the adaxial epidermis is initially also very fast, but at *t* = 60 s the kinetics of the contact angle change becomes similar to the reference measurement where the contact angle is changed purely by evaporation (**Figure** **6**, green curve). Even after 900 s, the drop on the adaxial epidermis showed a contact angle of Θ_AD,900s_ = 52 ± 18°.

By selectively coating the adaxial, abaxial, or both epidermises of an intact scale with PMMA, we were able to analyze the contributions of the epidermises to the hydraulic movement by comparison with unmanipulated scales. The angular changes of 12 scales were measured while increasing the humidity from 30% to 80% rh at 23 °C. The scales were allowed to equilibrate for 7 h. The uncoated scales showed unaffected bending deformation of *γ*_NoPMMA_ = 12.2 ± 4.2°. The rather high standard deviation results from the wide range of different sizes of the scales, which bend to different degrees. Six of these scales were coated on their adaxial epidermises, which resulted in just slightly lower angular changes of *γ*_AD PMMA_ = 11.5 ± 2.9°. The remaining six scales were coated on their abaxial epidermises, which entailed decreases of the angular changes to *γ*_AB PMMA_ = 8.5 ± 2.3°. All scales were then additionally coated on the formerly untreated side, respectively. With both sides being coated, the water absorption becomes very strongly reduced. The angular changes decreased to *γ*_Compl PMMA_ = 3.8 ± 2.5°.

**Figure 7:**
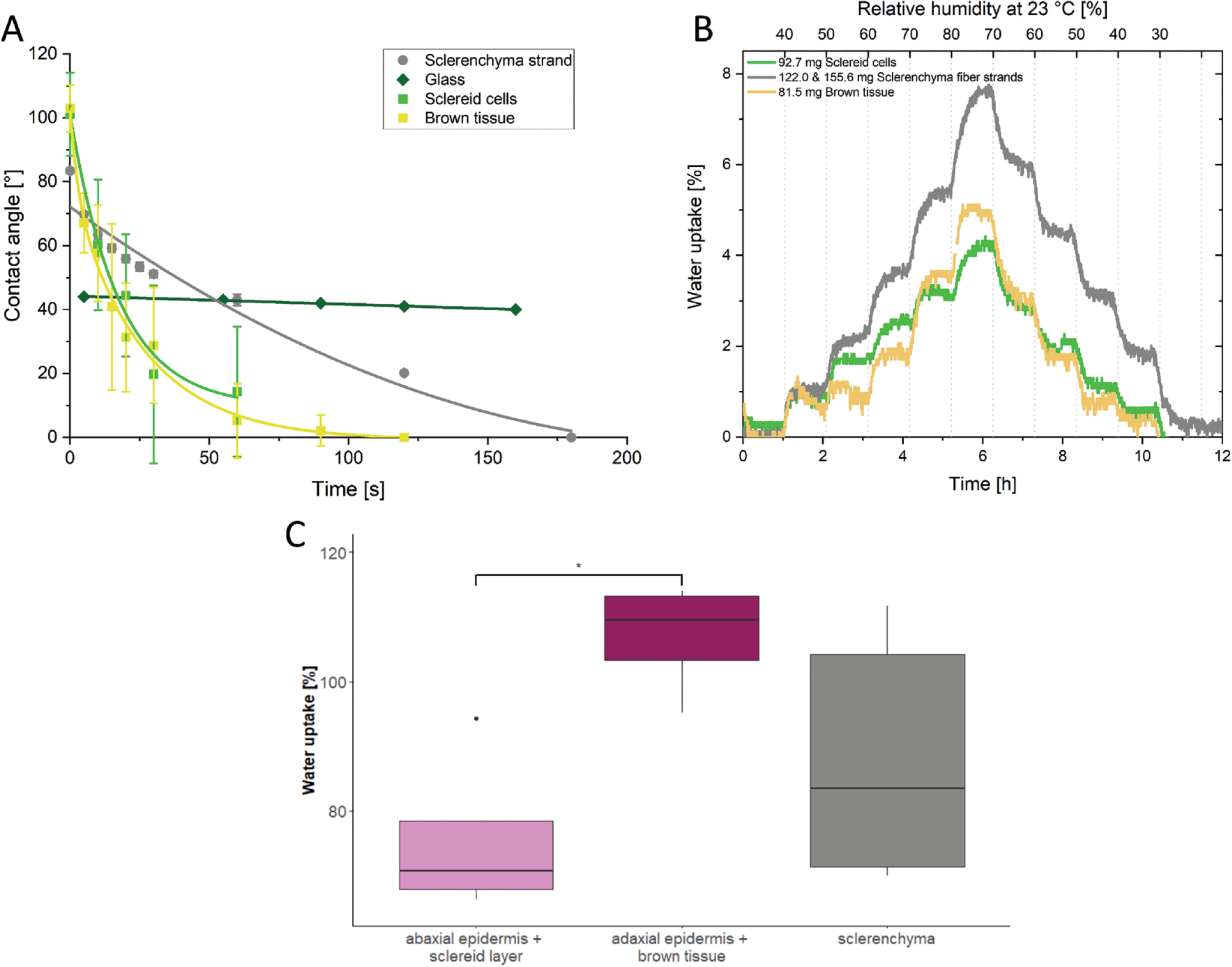
Hydration measurements with tissues from *P. wallichiana* scales. The contact angles of 5 µL sessile water drops on the sclereid layer and the brown tissue started with contact angles around 100° for both tissues (green and light brown). Already after 10 s, the contact angles were in the hydrophilic range and decreased rapidly. After 60 s, the contact angles of the sclereid layer could not be measured anymore due to swelling and deformation of the sample (green). The brown tissue absorbed the water drop after 120 s (light brown). The contact angle measured on the sclerenchyma fiber strands (gray) starts at 85° and decreases exponentially for 60 s and continues linear until the drop was completely spread and absorbed after 180 s *n* = 3 A). Water uptake as a function of time at 23 °C, while the rh is increased in 10% steps (top axis). Sclerenchyma fiber strands (156 mg) absorb 7.8 wt% water (gray) and the brown tissue (82 mg) takes up 5 wt% water (light brown) at 80% rh. Sclereids (92.7 mg) absorb 4.2 wt% (green). After each step, the weight reached a plateau indicating the reaching of the equilibrium. Given are mean values with standard deviation (whiskers) B). The water uptake of abaxial epidermis with sclereid layer (pink, *n* = 4), adaxial epidermis with brown tissue (purple, *n* = 4), and sclerenchyma fiber strands (gray, *n* = 5) were also obtained by weighing the samples in the dry and in the in fully water soaked state. The abaxial and adaxial tissue complexes showed a significantly different capacity to take up water, while the adaxial tissue was able to store more water than its own weight (110 wt%, median). The sclerenchyma fiber strands took up 83 wt% on average (median), Kruskal–Wallis test with subsequent Dunn's test, * = *p* < 0.05 C).

The kinetics of the contact angle development was also measured on the sclerenchyma fiber strands, the sclereid layer, and the brown tissue (**Figure** **7**A). To learn more about the pathway of water inside the scale, not just rate of water uptake, but also the extent of water absorption were considered. Therefore, the water absorption in rh was measured gravimetrically within 1 h (Figure 7B). The contact angle of the brown tissue (Figure 7A) started at Θ_BT,0s_≈ 100°. The contact angle decreased exponentially and the drop became absorbed within 120 s. Additionally, the brown tissue (82 mg, 23 °C, 80% rh) absorbed 5 wt% water (≈4.1 mg, Figure 7B, light brown). After each interval increase, the swelling of the brown tissue reached equilibrium after about 20 min. The contact angle of the sclereid layer (Figure 7A, green) also started around Θ_SC,0s_≈ 100° and also showed an exponential decrease indicating the absorption, but slightly slower than the brown tissue. However, through swelling and deformation of the sample, the contact angle could be measured only until 60 s after the drop deposition (Θ_SC,60s_≈ 18°). The sclereid cells (93 mg, 23 °C, 80% rh) absorbed 4.5 wt% (≈4.2 mg water, Figure 7B). The swelling also reached an equilibrium after about 20 min, but the equilibration time increased when the humidity was higher.

The contact angle of the sclerenchyma fiber strands (Figure 7A, gray) was much less hydrophobic and started at Θ_SF,0s_≈ 83°. While also a slow reduction of the contact angle was observed, after 3 min the drop became completely taken up by the tissue. This slower water uptake of a water drop is reflected also in the results of the gravimetrical measurement for the sclerenchyma strands (Figure 7B, gray). In these measurements they reached equilibrium slower than sclereid and brown tissue. The sclerenchyma fiber strands (≈140 mg, 23 °C, 80% rh) absorbed with ≈7.8 wt% (≈11 mg water) the most of all tissues in the scale, especially in the higher humidity range. Therefore, the sclerenchyma fiber strands required almost 1 h to swell to equilibrium. In contrast to this, all other tissues equilibrated quite fast, but took up only small weight percentages of water. Only the brown tissue more or less kept the same speed as it reached equilibrium, while also increasing the water uptake per step. It should be noted that during subsequent drying, the brown tissues´ and the sclereid cells´ weights decreased even further than the initial weight of the respective sample.

We additionally measured the water uptake of tissues completely submerged in water. Under these conditions, the abaxial epidermis with the sclereid layer increased 71 (+/− 10.4, *n* = 4) wt% in weight (on average (median) from 246 to 421 mg), while the adaxial epidermis combined with brown tissue increased its weight by 110 (+/− 9.9, *n* = 4) wt%, (from 143 to 290 mg on average (median), Figure 7C). The sclerenchyma fiber strands absorbed 83 (+/− 33, *n* = 5) wt% water, thus increased its weight from 141 to 288 mg on average (median).

### 3.3 Mechanical Properties

**Figure 8:**
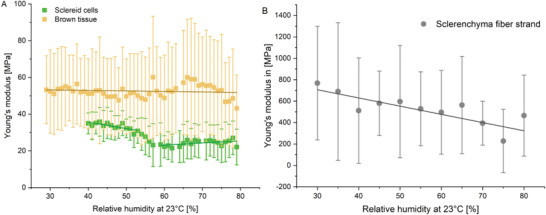
Mechanical properties of the individual tissues of the *P. wallichiana* pine cone scale. A) The Young's modulus of the sclereid cells (SC, green) is constant from 40% to 50% rh at 35 MPa. While increasing rh, the Young's modulus decreases to 25 MPa (from 50% to 60% rh at 23 °C) and stays constant at 25 MPa up to 80% rh. The Young's modulus of the brown tissue (BT, light brown) stays more or less constant at 52 MPa. B) Values for the Young's modulus of sclerenchyma fiber strands (SF, gray) decrease from 800 MPa at 30% rh to 200 Mpa at 75% rh. The Young's modulus was measured along the sclerenchyma fiber, respectively, fiber strand orientation. Given are mean values with standard deviation (whiskers).

Young's moduli were measured by AFM for all tissues along the sclerenchyma fiber, respectively, fiber strand orientation and as function of rh at 23 °C (**Figure** **8**). As expected for non‐homogeneous biological samples the moduli showed a strong variance of the individual values between the 64 locations measured for each sample. However, the average value showed a clear trend. The Young's modulus of the sclereid layer (Figure 8) was constant at low and high humidities, but underwent an almost step‐like decrease between 50% and 60% rh, from E_SC,50%_ = 35 MPa to E_SC,60%_ = 25 MPa. The brown tissue (Figure 8) possessed constant mechanical properties at E_BT,30 to 70%_ = 52 MPa until 70% rh was reached when it decreased slightly to E_BT,70%_ = 43 MPa. The sclerenchyma fiber strands, which have the highest—more than an order of magnitude higher—moduli among the cone tissues (Figure 8), decreased upon water uptake linearly from E_SF,30%_ = 800 MPa to E_SF,75%_ = 200 MPa.

### 3.4 Finite Element Simulation

**Figure 9:**
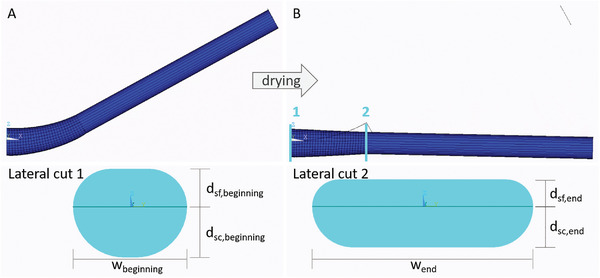
Schematic depiction of setting of the finite element simulations. The simulation started in the wet and bent state A). This angle was set as 0°. The simulated dry scale ended with an angular change of 24°. The cross section of the beginning of the bending zone (1) and the cross section of (2) are marked B). The cross section of the beginning of the bending zone has a *d*_sf,beginning_ = 0.6 mm thick layer of sclerenchyma fiber strands and *d*_sc,beginning_ = 0.8 mm sclereid layer in the dry and open state. The scale is *w*_beginning_ = 1.8 mm wide (lateral cut 1). The cross section of the end of the bending zone has a *d*_sf,end_ = 0.43 mm thick sclerenchyma strand layer and *d*_sc,end_ = 0.65 mm sclereid layer in the dry and open state. The scale is *w*_end_ = 3.5 mm wide (lateral cut 2).

Next, we performed a finite element simulation (**Figure** **9**), which allowed us to describe the Young's moduli of the sclerenchyma fiber strands and compare them to the experimentally resolved values, which were characterized by a high standard deviation. This way, situations that cannot be achieved experimentally, e.g., the contraction of the tissues during drying from 80% to 30% rh, could be simulated.

For the simulations, the scale was separated into two zones. In the first zone, the bending zone (Figure 1E), the sclerenchyma fiber strands are situated very closely to each other, so that almost no brown tissue is in between them. This assembly can be considered as one homogeneous layer. In contrast to the simple model of Timoshenko,^[^ ^6^ ^]^ the varying thicknesses of the sclereid and sclerenchyma layers and the varying widths were considered in the 3D model (Figure 9 lateral cuts). In the second zone, tissues do not contribute to the bending movement. Thus, between the end of the bending zone and the end of the scale, the structure was taken as completely straight (Figure 9B, everything to the right of cross section (2)). The wet state, i.e., the bent state, was set to be the starting situation (Figure 9A) and the drying of the scale was followed in the simulation (Figure 9B).

The simulation is based on the contraction of the sclereid layer of 22% observed in the µ‐CT measurement, while the sample was dried from being completely immersed in water to drying in air (in the simulation this was assumed as volumetric shrinking), and the measured Young's moduli of the sclereid layer. Furthermore, the shape of the scale in the wet state is set to 0° angular change and 0% contraction, and results in the dry state at *γ*_dry_ = 24.4° angular change and 22% contraction. (Please find the direction of contraction in section “3D scale architecture and tissue dimensional changes during actuation” above (after Figure 3).)

**Figure 10:**
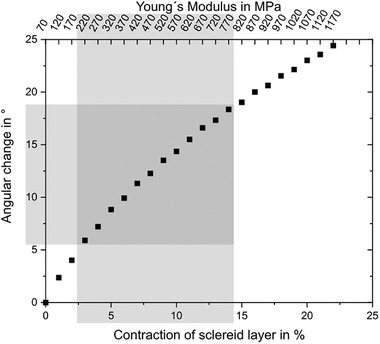
Results of the simulation of a scale of a pine cone as schematically depicted in Figure 9. The simulation of a scale bending during drying from the wet state with given angular change of *γ*_water_ = 24°, given contraction of the sclereid layer of ≈22% in length at the abaxial side and given values for the Young's modulus of the active layer shows a Young's modulus of the passive layer of 1170 MPa. After further assumptions, the measured angular change of 12° can be identified in drying from 80% rh (≈180 MPa) to 30% rh (≈ 800 MPa), which verifies the material measurements. In conclusion, while drying from 80% to 30% rh, the active layer contracts by roughly 11% and the Young's modulus of the passive layer decreases by 620 MPa.

The results show that the sclerenchyma fibers' Young's modulus had to be 1170 MPa to reach an angular change of 24.4°. Assuming that the contraction of the sclereid as well as the Young's modulus of the sclerenchyma fibers increase linearly when drying and a Young's modulus of 70 MPa when fully dried, **Figure** **10** shows the development of the angular change.

To verify the strong scattering measured Young's moduli of the sclerenchyma fibers between 30% rh (≈180 MPa) and 80% rh (≈800 MPa), the angular change resulting from simulations (Figure 10, highlighted in gray) is compared to the measured. Both the simulations and the measurements show 12° of angular change.

Therefore, it can be concluded that during drying from 80% to 30% rh the sclereid layer contracts by roughly 11% and Young's modulus of the sclerenchyma fibers increases by 620 MPa.

### 3.5 Force Measurements

Unmodified complete scales with a weight of 548 ± 0.06 mg developed a force of about 1.3 N (= 2.4 N g^−1^) on average during swelling and 0.9 N (= 1.8 N g^−1^) on average during drying.

**Figure 11:**
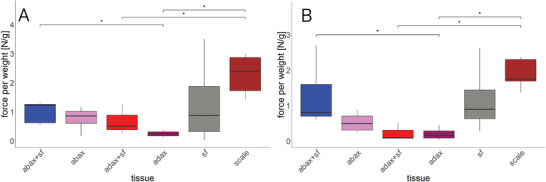
Forces generated by different tissues and tissue combinations taken from *P. wallichiana* cone scales. Forces generated by scales and tissues respectively tissue combinations during the uptake of water A). Forces generated during drying of scales, tissues and tissue combinations B). abax+sf = abaxial epidermis with sclereid layer and sclerenchyma fiber strands (*n* = 5), abax = abaxial epidermis (*n* = 4), adax+sf = adaxial epidermis with sclerenchyma fiber strands (*n* = 6), adax = adaxial epidermis with brown tissue (*n* = 4), sf = sclerenchyma fiber strands (*n* = 5), scale = whole scale (*n* = 5)), Kruskal–Wallis test with subsequent Dunn's test, * = *p* < 0.05.

In a series of similar experiments now the individual tissues were studied. The abaxial epidermis with sclereid layer and sclerenchyma fiber strands developed 1.2 N g^−1^ while swelling and 0.8 N g^−1^ while drying. In contrast to this, the same sample without sclerenchyma fiber strands developed 0.9 N g^−1^ while swelling and 0.5 N g^−1^ while drying. When now the adaxial epidermis was studied, the samples, where the sclerenchyma fiber strands were still attached develop 0.5 N g^−1^ while swelling and 0.2 N g^−1^ while drying. The adaxial epidermis with brown tissue generates 0.2 N g^−1^ while swelling and 0.1 N g^−1^ while drying. The sclerenchyma fiber strands developed 0.9 N/g both while swelling and drying (**Figure** **11**).

### 3.6 Dimensional Changes of Tissues and Strains on the Scale Surfaces During the Drying Motion

**Figure 12:**
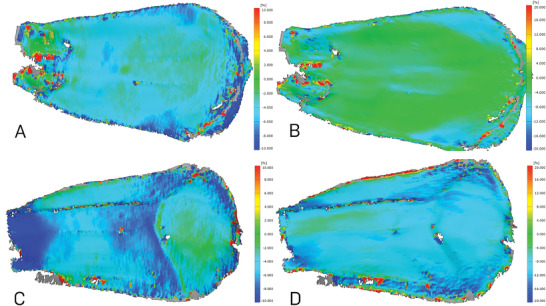
Strains on the abaxial and adaxial *P. wallichiana* scale surfaces during drying‐induced motion. Depicted are positive and negative strains along the longitudinal and transversal axis on the scale's abaxial A,B) and adaxial C,D) surface, which occurred during drying. Blue indicates negative strain (i.e., compression), while red indicates regions of positive strain (i.e., stretching) along the longitudinal A,C) and transversal B,D) axis. Note the altered legend scale for longitudinal and transversal strains.

Using 3D image correlation (3D‐DIC), recurring deformation patterns could be recognized in all investigated scales during drying‐induced motion. Due to the pronounced bending deformation, the apex of the scales is the region displaced the most. On the abaxial surface, mostly negative longitudinal strains occurred, which were concentrated in the very basal part of the scale (**Figure** **12**A) and decreased to −10% and more during the movement. On the adaxial surface, negative as well as positive longitudinal strains occurred (Figure 12C). Especially in the basal region, a few spots with positive strain could be observed during drying. In the time lapses, also the cross‐sectional bending prior to the actual opening bending could be observed (Video S1, Supporting Information). Transversal strains of about −8% occurred on the abaxial side of the scale (Figure 12B). The adaxial side showed almost no transversal strains (Figure 12D).

## 4 Discussion

With this work we could show that the structure of the bending zone is indeed to a certain extent comparable to a bimetal strip model as described by Timoshenko,^[^ ^6^ ^]^ but the whole bending mechanism of the scale is more complex than just one layer (active layer) expanding more than the other (passive layer) and thus forcing the system to curve. As a first aspect in the bending zone, the thicknesses of the tissues vary along the scale which makes the situation more complex. This is especially true for the actuating tissue, but it is also true for the composition of the passive layer composed of sclerenchymatous fiber strands and the brown tissue matrix. Furthermore, Young's moduli of the layers change due to water absorption, in particular the passive layer material becomes softer, so that the bending of the scale from dry to wet state is increased. On the other hand, the structural stiffness of the whole scale is reduced, when the scale dries and a backward bending of the scale, which is slightly curved in perpendicular direction in the wet state, occurs. These structural differences also lead to a difference in motion, from uniaxial bending to a more complex two‐phase bi‐axial motion.^[^ ^15^ ^]^

In the µ‐CT scans (see Figure 3), the individual tissues of a wet scale can be easily distinguished from each other. Thus, it is possible to reconstruct the exact inner structure of the entire scale at different humidity levels. With our analyses we are able to present detailed thickness profiles of these tissues in the whole scale. While the sclerenchyma fiber strands span nearly the entire scale, sclereid cells are only located in about 2/3 of the scale. Both tissues are thickest in the basal region of the scale and thinner toward its apical end. We suppose that this causes the strong bending in the basal region, while the deformation of the apical end is geometrically amplified through the length of the scale.

The sclerenchyma fiber strands were found to also increase to some extent in length during wetting. This is most probably due to their fibrous structure and indicates an expansion to water absorption with the 3rd root of the volumetric water uptake. The sclereid cells, however, predominantly elongate in length and only very little in the other two dimensions. This is caused by a natural smart design of cellulose fibers in the sclereid cell wall, which are wrapped around the cell body like a corset.^[^ ^3^ ^]^ This corset prohibits an expansion perpendicular to the fiber axis and directs the whole swelling process into a length expansion. The expansions of the individual tissues measured during wetting fall well in the range known from other plant tissues of comparable structure and are significantly larger than those of the sclerenchyma fiber strands.^[^ ^19^, ^20^ ^]^

An important question is how the water enters the scale. The outer surface of the scale consists of epidermises, that control the water access to the inner tissues. The adaxial and abaxial epidermises showed different kinetics in water uptake. The results of the measurements where access to one side of the scale is blocked suggest a faster water penetration through the abaxial side of the scale: A strongly retarded water uptake through an impermeable PMMA coating deposited on the adaxial epidermis and the fast water absorption through the abaxial epidermis (where the sclereid layer is located) resulted in only a minimally reduced bending of the scales (from 12.2° to 11.5°). The same coating deposited on the abaxial epidermis, however, markedly decreased the bending angle from 12.2° to 8.5°, meaning that the water uptake through the “preferred” way is prevented. However, some water still enters through the adaxial epidermis albeit at slower rate. The completely coated scales still bent by 3.8°, despite the water absorption is nearly prevented. In nature, the *P. wallichiana* cones hang on the trees and the abaxial sides of the individual scales face out‐ and upwards. Therefore, the setup of the whole cone determines where water, e.g., rain, can get in contact with the epidermises. In the open state, the apophysis, the strongly lignified tip of one scale shields the adaxial epidermis of the scale underneath like an umbrella, while humidity still can reach both epidermises. While both sides are accessible to rain in the open state, only the abaxial epidermis is accessible in the closed state. We suppose that this is the reason why the opening motion starts at the cone base, while the closing motion is a homogeneous motion of the whole cone.

**Figure 13:**
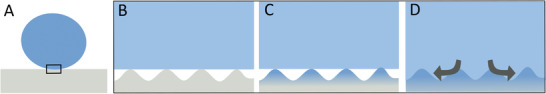
Simple schematic representation of the wetting behavior of water on a pine cone scale's epidermis. The drop A) sits on the surface structures of the adaxial epidermis B). After some contact time, the structures start to absorb water and thus increase the hydrophilicity C). Due to the increased hydrophilicity of the structures, the drop sinks in the structures and undergoes a transition from Cassie–Baxter wetting to Wenzel wetting D).

The water uptake also influences the contact angle, i.e., the wetting of the scale/cone with a bulk water drop. At the very beginning, the deposited drop rests on the roughness features of a rather hydrophobic surface, which allows a drop to easily roll off from a hanging cone (see Figure 6). This roll‐off reduces water contact and thus avoids that the cone opens and closes upon contact with a few sprinkles of water. However, when water stays in contact with the cone, within about 30 s of contact, a drop of water situated on the abaxial epidermis sinks into the surface and is taken up by the scale tissue. The abrupt change of the drop shape might be explained by a Cassie Baxter to Wenzel transition^[^ ^20^ ^]^ where the drop sinks into the grooves of the epidermis (**Figure** **13**) and no air pockets remain under the droplet. Such a transition might be due to the swelling of the epidermis (see Figure 2E,F), which increases the surface energy and thus makes the surface better accessible for water. As the wetting behavior is correlated with the water uptake and accordingly swelling kinetics, this process takes some time. On a rough, now hydrophilic surface the drop readily spreads.

The whole process induces a behavior that a drop upon initial contact rolls off rather easily. Only when liquid water is present on the cone for longer periods of time water spreads on the surface and induces hydromorphic behavior. In conclusion, first, both sides of the scale (adaxial and abaxial) behave similarly. After a couple minutes, when liquid water has spread in the abaxial epidermis, the abaxial side takes up the drop within a few seconds due to a difference in the swelling kinetics, while for the adaxial epidermis the change in contact angle is still rather slow.

The hydration measurements and mechanical analysis of the tissues allow to propose the further pathway of water inside the scale (see Figure 7A,B): We found that the sclereid layer takes up water from air faster than the sclerenchyma fiber strands, however, the water uptake is only rather small (up to 4 wt%). Due to this water uptake in humid environments, the Young's modulus is decreased by ≈10 MPa (see Figure 8). The water uptake of the sclereid layer is, therefore, rather small, although it consists of cells with thick cell walls and many intracellular spaces (Figure 2A). When immersed in water, the weight of the abaxial epidermis with sclereid layer increased by 71 wt% (see Figure 7C). This stronger weight increase, if soaked in water presumably results from filled intercellular spaces, which we presume to increase the swelling pressure and, therefore, to speed up the motion. In high humidity, these intercellular spaces are not filled, since the humidity only adsorbs into the cell walls. Therefore, osmotic pressure and stronger swelling in water lead to a more pronounced motion.

In contrast to this, the sclerenchyma fiber bundles can absorb 7.8 wt% (see Figure 7A,B) water in humid environments, which is almost three times the amount of water that the sclereid layer takes up in humid air. When immersed in water, the sclerenchyma fiber bundles absorb 83 wt%. During water absorption the Young's modulus of this layer decreases by 75% down to 200 MPa, though with a high standard deviation (see Figure 8). This strong water absorption and strong decrease in Young's modulus of the sclerenchyma fiber strands is surprising, because each strand consists of a densely packed bundle of fibers (Figure 2B).

The brown tissue absorbs a small water droplet the fastest (120 s, see Figure 7A) and increases its weight even 1 wt% more than the sclereid layer in humid air (5 wt%, see Figure 7B), but decreases its Young's modulus the least, from 55 MPa at 30% RH and 23 °C only by 10% to 50 MPa at 80% RH and 23 °C (see Figure 8). The adaxial epidermis with brown tissue increases its weight by 110 wt% in water. Both, the fast water absorption from humid air and the strong water absorption in liquid water can be explained by its friable structure (Figure 2C). The porous structure can absorb water fast due to the high surface area.

In summary, our experiments show that the abaxial epidermis of the scale is clearly the interface through which the predominant part of water displacement for scale actuation takes place. Though, abaxial tissues (abaxial epidermis + sclereids) are not as capable of storing water as, e.g., brown tissue or sclerenchyma fiber strands.

The decreased weight after gravimetric measurement probably comes from losing some included water which has more time upon drying to migrate to the surface and to evaporate. It could also be, that some volatile molecules are dissolved during swelling and then evaporate together with the water. Both would lead to a decreased weight compared to the initial weight after performing the gravimetrical measurements (Figure 7B).

The water uptake of submerged tissues showed the same behavior as water uptake by moisture (see Figure 7C). The abaxial epidermis with sclereid layer absorbed slightly more than half of the water as absorbed by the combined adaxial epidermis and brown tissue. The water uptake of the sclerenchyma fiber strands varied strongly but the values are roughly in between those of the two epidermises. The difference in water uptake of the epidermis is statistically significant (Kruskal–Wallis‐test, n per tissue = 5, chi‐squared = 6.5473, df = 2, *p* = 0.03787).

The finite element simulation based on the contraction of the active sclereid layer as well as the Young's modulus data agree well with the proposed water uptake‐model. The simulated bending zone is considered as a 3D structure with two different stacks associated with the two layers, i.e., the active and passive layer. This procedure and model deviate from the bilayer‐model proposed by Timoshenko,^[^ ^6^ ^]^ as the included assumptions and simplifications there do not comply with the oval cross section of the simulated pine cone scale and to the fact that the width as well as the thickness of the layers vary over the length of the scale (Figure 9, lateral cuts 1 and 2). Since the sclerenchyma fiber strands are so close together that almost no brown tissue is in between (see bending zone, Figure 1D,E), it is considered as one single layer without discrete modeling of the strands. The sclereid layer in the simulation contracts according to the µ‐CT scans and the Young's modulus of the sclereid layer changes according to micro indentation measurement. The Young's modulus of the sclerenchyma fibers as well as the contraction of the sclereid layer could be fit to the experimentally determined data very well by the simulations. However, the bilayer simulation lacks the complex structural composition of the scale, especially the epidermis, which regulates the water uptake, so that the assumption of volumetric swelling or shrinking being constant over the sclereid layer thickness is in reality not met. Furthermore, the two‐phase motion with a slight additional bending in perpendicular direction^[^ ^14^ ^]^ was neglected in order to keep the simulation simpler.

The developed force of the whole scale and different combinations of tissues helped understanding the importance of the structural integrity of the whole system, i.e., the scale (see Figure 11). We found that whole scales were able to produce highest forces. The second efficient force generator was the combination of abaxial epidermis, sclereid layer, and sclerenchyma fiber strands, which basically represents the long proposed bilayer‐model.^[^ ^3^ ^]^ This shows impressively, how nature is able to improve performance by elaborating on simple concepts, in this case through additional materials with specific properties, like brown tissue and the epidermises on the ab‐ and adaxial side. Furthermore, also single tissues are able to perform bending, especially the sclerenchyma fiber strands with the sclereids and brown tissue. The bending of sclerenchyma fiber strands and sclereids complement each other to create a synergistic, forceful movement.^[^ ^9^ ^]^ Brown tissue in combination with sclerenchyma fiber strands as well as sclereids alone were not able to create noticeable movements or forces. The fact that even several single tissues perform movements reminds of shape memory materials,^[^ ^22^ ^]^ but may in fact be due to gradients in tissue composition as well. Surprisingly, if all the forces of each tissue of one scale are added up and compared to the forces developed by a whole scale, the force while swelling is just 28% and while drying 31% of the force of the whole, unmanipulated scale, respectively. When normed to its weight, the tissues produced 82% (swelling) and 89% (drying), respectively, of a whole scale. It should be noted, that some tissue was lost during preparation of the samples, even though one has taken care to minimize this loss. However, we believe that the majority of the force loss is due to the loss of scale integrity and hierarchical structuring. The interconnection between the tissues and the cohesion through the epidermis have a supposedly great potential to increase the generated forces of the individual tissues in the whole system.

The 3D digital image correlation confirmed the results from our µ‐CT measurements as well as from our simulations, as we found positive and negative strains distributed stochastically on the ab‐ as well as adaxial surface of all investigated scales, while stretching and compression only occurred in the very basal region, described as bending zone. While the bending zone is compressed abaxially during drying, the adaxial site shows spots of strong stretching (Figure 12). This distribution is in accordance with the idea of an elongated lever, actuated by a small bending zone as described above. We expect the slight compression visible on both surfaces to be a result of the drying, leading to a shrinkage of the material. Also the calculated alteration of the sclereid tissue of 11% necessary for the bending movement approximately fits the shortening observed in our µ‐CT analyses (18%).

The motion of the individual tissues, measured by Quan et al., are consistent with the force measurements we have performed simultaneously with the individual tissues. Quan et al. concentrated their investigation on the varying structure of the sclereid layer and the motion in water, while we concentrated more on the hydration of the tissues and the structural and mechanical changes upon water absorption. However, we agree with the overall conclusion that the structure of the pine cone is more complex than the previously reported bilayer setting based on Timoshenko's bimetallic strip model.^[^ ^6^, ^9^ ^]^ However, our simulation is designed as a bilayer because the bending zone where the main bending occurs is very close to a real bilayer structure. Moreover, we did not assume a constant thickness of the double layer, but represented the geometry of the scale tissue in more detail.

**Figure 14:**
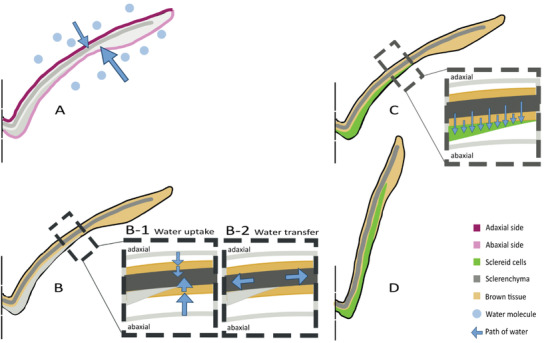
Model of water uptake of a pine cone scale. First, the water molecules enter faster on the adaxial side (A, pink). Second, the brown tissue and the sclerenchyma fiber strands take up water B‐1). The locally increased pressure of the swollen sclerenchyma fiber strand and brown tissue around is expected to induce a passive water transfer to the rest of the scale B‐2). Also, the fiber strands are getting soft and therefore allow and support the bending. Third, the water reaches the sclereid layer C). The sclereid cells expand longitudinal. This volume increase finally causes the scale to bend D).

Considering all the described measurements, our model (**Figure** **14**) proposes a description of the path of water in the scale. The contact angle measurement of the epidermis showed a faster uptake at the abaxial side of the scale. Therefore, the water is expected to be mostly absorbed at the abaxial epidermis (Figure 14A). From there, the water is passively distributed to all three tissues, i.e., the sclereid layer, the brown tissue and, embedded in the brown tissue, the sclerenchyma fiber strands. The brown tissue exhibits the fastest water uptake and is therefore expected to absorb first. The water uptake of the brown tissue is the fastest and is therefore expected to absorb first, followed by the sclerenchyma fiber strands, which also absorb a major fraction of the water and softens, thus enabling and amplifying bending (Figure 14B). The tension forces generated by the swelling of the sclerenchyma fiber strands are supposedly damped by the friable structure of the brown tissue. These insights can be merged into a thought experiment: If we have two components, A and B, in a closed system and component A swells (and therefore increases its volume), the other component B is compressed. The increased pressure on component B reduces its water absorption capability, at least in the places where component A is already swollen. However, this also introduces the possibility of passive transport through the pressure gradient at locations where component A is not yet swollen. This allows a uniform swelling of component A. In this model, component A represents the sclerenchyma fiber strands and component B the brown tissue. Therefore, the brown tissue contributes significantly to the distribution of water in the scale. Previously, the sclerenchyma fiber strands have been described in the literature as a purely passive layer, which is defined as being nonresponsive to the trigger, and the brown tissue has not been mentioned at all. The results obtained here indicate, however, that the sclerenchyma fiber strands and brown tissue are equally essential tissues for the movement of the scale due to the strong water absorption and softening properties.

Next, the sclereid layer absorbs water (Figure 14C). With the passive layer being soft and deformable and the active layer expanding strongly, the scale bends upward and closes the cone (Figure 14D). The lateral bending of the scale in direction to the adaxial side ensures a closed packaging of the cone. In addition, we expect that the combination of short and thin sclerenchyma fiber strands and comparatively more sclereid in the lateral periphery of the scale, might be the reason for the change in curvature of the cross section upon drying/swelling.

## 5 Conclusion

Pine cone scales bend because of a complex interaction of different tissues, each of which makes a contribution to the movement. We studied the kinetics of wetting, gravimetric water uptake, the Young's modulus as a function of humidity, performed 3D digital image correlations and measured the forces of several tissues and tissue combinations. These results enabled us to establish a detailed model on the water uptake and distribution in the pine cone (scales) of *P. wallichiana*, which extends the simple bilayer model of Timoshenko^[^ ^6^ ^]^ that has been used to describe the bending motion so far. The water uptake mainly happens through the abaxial epidermis of the scale.

As the surface is initially rough and quite strongly grooved, a contacting water drop is in the Cassie–Baxter state with air pockets in the grooves below it. This causes a drop, which comes only into brief contact with the scale, to roll off. Upon prolonged water contact the surface layer begins to swell, the contact angle on the rough surfaces is decreased causing a transition between Cassie–Baxter wetting to Wenzel wetting. This allows then water to spread on the scale surface. The water is there taken up by the brown tissue and the sclerenchyma fiber strands, which also distribute the water in the scale. The sclerenchyma fiber strands soften thereby strongly. After the water is absorbed by the sclereid layer, it expands in longitudinal direction, because it is hindered to expand in other directions by a stiffening cellulose “corset” in the cell walls. The sclerenchyma fiber strands allow and amplify the bending through the softening (bottle neck of motion) and the sclereid layer executes the bending through the expansion. In contrast to the conventional bilayer model, which consists of a water‐swellable (“active layer”) and a not or much weaker swellable layer (“passive layer”), the cone scale consists of several layers, which all take up water. Upon water uptake, the sclerenchyma fiber strands soften strongly and thereby “unlock” the bending movement, which is then initiated by the 1D swelling of the “corset‐harnished” cells in the sclereid layers. Hence, by implementing these different tissues in one and the same process, i.e., swelling with water, the “unlocking” of the layers and bending is achieved. An additional feature is that, although the materials used in constructing the tissues are chemically identical, their arrangement varies along the scale, so that the scale can be described as consisting of a bending zone and a “flap‐like” layer, which does not contribute to the bending process in any major way but is moved almost exclusively passively. With this better understanding of the bending mechanism, we will be able to develop better technical systems for hygromorphic structures based on this biological inspiration.

## Conflict of Interest

The authors declare no conflict of interest.

## Supporting information

Supporting Information

Supporting Video 1

Supporting Video 2

## Data Availability

The data that support the findings of this study are available in the supplementary material of this article.
