# Supplementary material for: The Structural and Mechanical Basis for Passive‐Hydraulic Pine Cone Actuation
Source: Adv Sci (Weinh). 2022 May 14;9(20):2200458. doi: 10.1002/advs.202200458 (PMC9284161; doi:10.1002/advs.202200458)
Supplement: Supplementary file 1 — Supporting Information [file ADVS-9-2200458-s003.pdf]

ADVANCED SCIENCE
Open Access

# Supporting Information

for *Adv. Sci.*, DOI 10.1002/advs.202200458

The Structural and Mechanical Basis for Passive-Hydraulic Pine Cone Actuation

*Carmen J. Eger, Martin Horstmann, Simon Poppinga, Renate Sachse, Rebecca Thierer, Nikolaus Nestle, Bernd Bruchmann, Thomas Speck, Manfred Bischoff and Jürgen Rühe**

# Supplementary
# The Structural and Mechanical Basis for Passive-Hydraulic Pine Cone Actuation

C. J. Eger[1,3], M. Horstmann[2], S. Poppinga[2,3,4], R. Sachse[5], R. Thierer[6], N. Nestle[7], B. Bruchmann[7], T. Speck[2,3], M. Bischoff[5], J. Rühe[1,3]

1 University of Freiburg, Department for Microsystems Engineering, Chemistry and Physics of Interfaces
2 University of Freiburg, Botanic Garden, Plant Biomechanics Group
3 Cluster of Excellence livMatS @ FIT – Freiburg Center for Interactive Materials and Bioinspired Technologies, University of Freiburg, Germany
4 Technical University of Darmstadt, Department of Biology, Botanical Garden
5 Technical University of Munich, School of Design and Engineering, Institute for Computational Mechanics
6 University of Stuttgart, Institute for Structural Mechanics
7 BASF SE, Ludwigshafen, Germany

Classification: Research Article

Keywords: pine cone movement – kinematical and structural analysis – hydration measurement – tissue mechanics – µ-CT scans – finite element simulation – model for water absorption

## Supplementary

| Measurement | Additional information to measurement | cones | scales |
|---|---|---|---|
| Scale motion | | 4 | 12 |
| SEM | | 1 | 1 |
| structural analysis via microscope | | 1 | 4 |
| Contact angle measurement | | 2 | 4 |
| Gravimetric measurement | | 2 | 3 |
| AFM | | 1 | 1 |
| force and gravimetric measurements | whole scale | 2 | 5 |
| | abaxial tissue | 2 | 4 |
| | abaxial + sclerenchyma | 2 | 5 |
| | sclerenchyma | 2 | 5 |
| | adaxial + sclerenchyma | 2 | 6 |
| | adaxial tissue | 2 | 4 |
| µCT | P. sylvestris (length of sclerenchyma measured at 9 fibres, tissue swelling at 3 sites of the µCT-sample) | 1 | 1 |
| 3D-DIC | | 2 | 9 |

http://public.cpi.imtek.uni-freiburg.de/video-aufbau-schuppe-uct.mp4

**Three-dimensional reconstruction of the scales' tissues.** The video shows the various tissues observable in a *P. sylvestris* scale: The outermost layer comprises mostly epidermal layers and adjacent material with similar properties when analyzed with a µCT-scanner. Brown tissue is located around the cable-like sclerenchymatous tissue. The hygroscopically most active sclereid layer is located abaxially of the sclerenchymatous tissue.

http://public.cpi.imtek.uni-freiburg.de/aramis-4-fold.mp4

**Three-dimensional digital image correlation of a *P. wallichiana* scale.** Upper videos show the displacement occurring during the opening motion on the abaxial (left) and adaxial (right) sides. Scale tips are displaced the most due to angular amplification, while basal regions remain almost unaltered. Lower videos show the strain (in %) occurring in longitudinal direction, again on abaxial (left) and adaxial (right) sides. While especially the basal region of the abaxial side is characterized by negative strain, spots in the basal region of the adaxial side indicate positive strain during the drying-induced motion, which both correlates with our observation that most hygroscopic material is located basally.

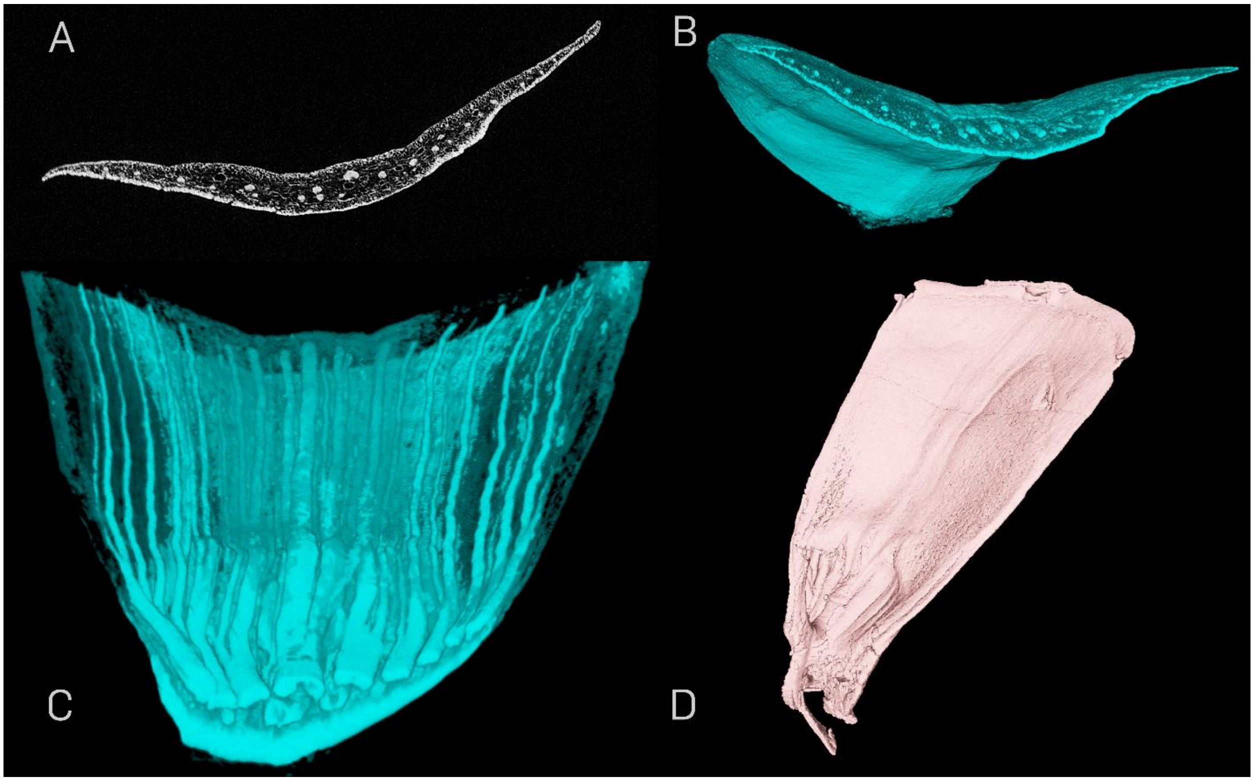


*Figure 1:* ***P. wallichiana µCT dataset.*** *Shown are a digital cross section (A), a combined cross section and view from the abaxial side (B), a combined cross section at the base and an opaque view from the adaxial side, showing the sclerenchyma fibers respectively fiber strands (C), and a lateral view on the reconstructed scale model (D).*
